# Behavior change techniques in mobile health interventions promoting recovery from substances: A synthesis of reviews and meta-analyses

**DOI:** 10.1177/20552076261458891

**Published:** 2026-06-04

**Authors:** Nithya P. Gurumurthy, Lex Hurley, Brooke T. Nezami, Nisha Gottfredson O’Shea

**Affiliations:** 1 Gillings School of Global Public Health, 2331 The University of North Carolina at Chapel Hill, Chapel Hill, NC, USA; 2 School of Medicine, 2331 The University of North Carolina at Chapel Hill, Chapel Hill, NC, USA; 3 Department of Health Behavior, 2331 The University of North Carolina at Chapel Hill, Chapel Hill, NC, USA; 4 Department of Nutrition, 2331 The University of North Carolina at Chapel Hill, Chapel Hill, NC, USA; 5 6856 Substance Use, Prevention, Evaluation & Research, RTI International, Research Triangle Park, NC, USA

**Keywords:** substance use, alcohol, tobacco, illicit drug, mHealth, mobile health, JITAI, BCT, behavior change techniques

## Abstract

**Objective:**

This synthesis of reviews and meta-analyses delves into the landscape of behavior change techniques (BCTs) employed in digital interventions designed to help individuals abstain from or reduce consumption of substances (including alcohol, tobacco, and illicit drugs). This review considers the “black box” problem in mHealth programs by using Michie et al.’s BCT taxonomy to describe BCTs that have been used in intervention literature and to explore potential active ingredients that may contribute to intervention effectiveness.

**Methods:**

We synthesize findings from 49 systematic reviews and meta-analyses. While individual studies often express inconclusiveness for the effectiveness of specific BCTs, this review uncovers promising avenues for future research. Our analysis focuses on mobile health (mHealth) just-in-time adaptive interventions (JITAIs), with a specific emphasis on substance use reduction.

**Results:**

Eleven BCTs were studied extensively in these reviews, including self-monitoring of behavior, feedback on behavior, goal setting, social support, prompts/cues, and behavior substitution. Our synthesis of evidence points to prompts/cues as particularly promising and highlights a handful of BCTs that demand further investigation, including self-monitoring, goal setting, and feedback on behavior.

**Conclusions:**

This review identifies specific limitations in each step of review formulation and provides nuanced suggestions to enhance the efficacy of future research endeavors.

## Introduction

Although addiction is treatable, individuals in recovery typically experience multiple lapse and relapse episodes. According to the National Institute on Drug Abuse, 40 to 60% of patients with substance use disorders (SUDs) relapse after treatment.^1^ Relapse can be dangerous or even deadly for some drugs such as opioids and alcohol, as persons in recovery can more easily overdose due to lack of tolerance following a period of abstinence.^1^

The most effective behavioral interventions for relapse prevention include cognitive behavioral therapy for addiction and co-occurring mental health disorders, contingency management, and mindfulness coaching.^2^ The standard treatment for SUDs involve medication management (when available) and monthly individual counseling, usually with a 3- or 6-month follow up.^3,4^ Although the standard treatment methods are effective, individuals with SUD face many barriers to in-person treatment, and access to counseling may not be readily available in the moment when an individual is facing strong urges to use; recent estimates suggest that only 10% of individuals with an SUD are receiving treatment.^2,5^ Barriers include perceived stigma, fear of punishment, lack of social support, lack of trust in providers, transportation difficulties, and lack of available services.^6^ Substance use interventions can also be applicable for the large proportion of individuals who struggle from SUD but do not have a formal diagnosis, or whose symptoms are subclinical.^5,7^ Thus, for the remainder of this review, we will refer to “substance use involvement” or “substance use recovery,” instead of “substance use disorder” to be inclusive of the more general population affected by substance use problems.

Just-in-time adaptive interventions (JITAIs) are a promising mobile health (mHealth) methodology for complementing or replacing in-person behavioral therapy that is either out-of-reach or undesired, as JITAIs focus on providing an individual with tailored support, at the right time and right level.^8^ Generally, just-in-time components of mHealth interventions are delivered through program-initiated push notifications or text messages in the moment when an individual is likely to need immediate support. In some cases a program could apply “just-in-time” pull components (i.e., participant-initiated) if some type of support is immediately available to an individual that is seeking help in a time of need (e.g., pressing a “support” button in an app or texting an alert line).^8^ JITAIs have been used to target behaviors such as reducing sedentary behavior, increasing physical activity, and preventing dietary lapses, in addition to smoking and substance abuse.^8–11^ These interventions may be particularly helpful for relapse prevention because JITAIs are uniquely positioned to target predictors of inhibitory self-control to support abstinence when cravings are strong.^8^

JITAIs, which are relatively new, are becoming increasingly popular and well-studied. The number of studies indexed in Google Scholar using search terms “‘JITAI’ AND ‘mHealth’” have steadily increased from 74 published in 2019 to 275 published in 2025. However, most of them use many treatment components simultaneously (e.g., web- or app-based lessons together with standardized push messages and a social interaction component). The use of simultaneous intervention components is natural and mirrors most health promotion programs, whether delivered in person or via remote methods. Nevertheless, the use of multiple components and behavioral strategies simultaneously, particularly when delivered using a novel modality, produces a “black box” problem wherein it is difficult to evaluate which strategies are driving the behavior change.^12^ As the popularity of JITAIs continues to grow, it is critical to understand the differential efficacy and effectiveness of individual mobile intervention components.

Michie et al.’s Behavior Change Technique (BCT) taxonomy is a set of 93 BCTs that enables researchers to identify specific components and applications in interventions as “active ingredients” of an intervention.^13^ The application of BCTs in non-mHealth interventions targeting substance use have been found effective in several studies.^14^ Incorporating specific BCTs, such as problem-solving, goal setting of behavior, self-monitoring of behavior, and biofeedback, have led to reductions in substance use in traditional interventions, supporting the utility of this taxonomy in identifying active ingredients of interventions.^15,16^ The identification of individual BCTs within interventions can allow for future disentanglement of treatment component effectiveness and lead to the development of more efficient and effective interventions.

To fill this gap in the literature, this synthesis summarizes existing systematic reviews and meta-analyses that have sought to examine the role of BCTs in digital behavior change interventions for substance use recovery.

## Methods

As part of a larger systematic review and meta-analysis, we searched PubMed, Web of Science, Scopus, and Embase using MeSH and Boolean search terms to identify eHealth and mHealth studies with at least one just-in-time (JIT) component targeting a reduction in substance use behaviors or dietary behaviors such as calorie restriction. The full search terms applied are located in Appendix A. This review of reviews represents a sub-analysis of the review articles identified with this search; these articles were omitted from the larger systematic review and meta-analysis project. Here, we examine systematic reviews and meta-analyses published between 2009 and 2024 returned using our search terms, focusing on the reviews of substance use (i.e., reviews about dietary behaviors were excluded for the purposes of this review paper).

As part of the larger study, two reviewers screened the titles and abstracts of each paper identified using search terms; all systematic reviews and meta-analyses were moved to a separate collection to be used for this synthesis of reviews. These review papers were then fully reviewed by two reviewers, and those that fit inclusion criteria were included in this paper. Inclusion criteria for this review included: must be identified as a systematic review, meta-analysis, or review of more than one quantitative study that contained at least one JITAI component; must target outcomes related to substance use (i.e., alcohol, tobacco or nicotine, or other illicit drugs); describes at least one clearly identifiable BCT from Michie et al.’s BCT taxonomy in its content either directly referenced within the text, or implied in the text and identified by our team. For inclusion in this synthesis, reviews were required to focus on interventions in which all primary studies incorporated a JITAI component, although specific implementation and features of the JITAI varied across studies.

In some cases, BCTs were directly named in the review papers, and these were recorded; however, for other reviews, BCTs were described in the text, and our team identified which BCT was being referenced based on operational definitions outlined in Michie et al. (2013). For example, in several studies, “self-monitoring of behavior” was not directly named, but “self-reporting” was described and BCT [2.3] was identified. For example, interventions that required participants to record or track their substance use behaviors were coded as *self-monitoring of behavior* [2.3]; interventions that provided participants with personalized information about their behavior were coded with *feedback on behavior* [2.2]; interventions that required participants to establish goals or targets for their behavior changes, either long-term or short-term were coded as *goal setting (behavior)* [1.1]. For some BCT categories, particularly *social support* [3.1-3.3], reviews often evaluated the concept at a broader level, rather than distinguishing between specific subtypes. In this case, we also synthesized findings, grouping its use under the broader “social support” category. Once reviews were identified for inclusion, each review was evaluated for which BCTs were included, whether the unique effects of each BCT were able to be isolated given the study design, which BCTs were found effective, and any other notable conclusions.

For each included review, we extracted which BCTs were present, whether their independent effects could be isolated, and any reported conclusions regarding effectiveness. Effectiveness classifications were based on the conclusions reported by the authors of each review. In this synthesis, a BCT was considered “effective” if the review authors reported that it was associated with positive behavioral outcomes. This synthesis reflects the determinations made by the review authors, rather than applying a new effectiveness criterion. Some reviews did not explicitly evaluate or declare the effectiveness of the individual BCTs, often focusing on the effectiveness of the intervention instead. For these reviews, effectiveness was coded as “N/A,” but they were still included, as they provide information on which BCTs were implemented in digital interventions, even if their independent effectiveness was not determined.

Extracted data was coded manually using a structured Excel spreadsheet using a predefined framework If a review reported that the unique effect of a BCT was evaluated but not found effective, it was coded with 0; if a BCT was evaluated and found effective, it was coded with 1; if a BCT was evaluated but results were mixed between studies, it was coded with 2. As noted previously, if the unique effect of the BCT could not be evaluated, results were coded as “N/A”. “Mixed results” are defined as reviews that included at least one study that found the BCT effective for decreasing the likelihood of substance use involvement, while other studies in the same review found that BCT to have null effect on substance use involvement.

“Positive behavior change” or “positive clinical outcomes” refers to the reduction of substance use involvement, including but not limited to smoking and alcohol use reduction. These outcomes were used to inform effectiveness classifications in this synthesis.

## Results

### Summary of search results

**Figure 1.**
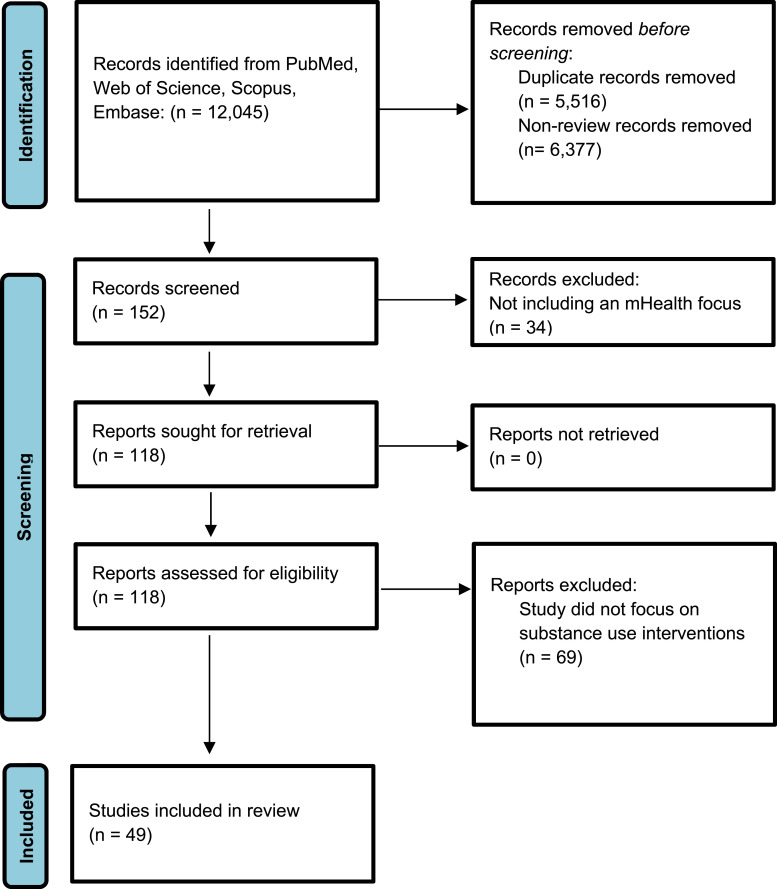
PRISMA flow diagram of study selection process.

Of the N = 12,045 articles retrieved in the initial search, we identified 152 systematic reviews and meta-analyses which could facilitate analysis of differential effectiveness of BCTs across included digital interventions. After removing duplicates as well as those that did not contain an mHealth or JIT component, 118 systematic reviews and meta-analyses remained. Of these reviews, n = 49 included substance use outcomes as detailed in the PRISMA flow chart in Figure 1. Intervention modalities covered in reviews included smartphone apps, reviewed in 21 reviews; text messaging, in 31 reviews; websites or web-based interventions, in 21 reviews; wearable technologies, in 3 reviews; and video games, in 3 reviews.

### Behavior change techniques identified

Eleven primary BCTs were found in the 49 reviews that studied substance use, summarized in Table 1 (BCT names are *italicized* and numbers are identified in brackets). The eleven most widely used and studied BCTs in mobile health interventions include *self-monitoring of behavior* [2.3] (40 reviews^17–55^), *feedback on behavior* [2.2] (34 reviews^17,18,20,21,23,26–34,36–38,40,41,44–51,53,56–61^), *goal setting (behavior)* [1.1] (23 reviews^17,18,23,26,28–32,35,40,45–47,49–54,61^), and types of social support [3.1, 3.2, 3.3] (13 reviews^17,23,26,29,31,32,35,52–55,58,62^). Other commonly used BCTs include *problem solving* [1.2] (7 reviews^18,23,26,32,44,50,55^), *action planning* [1.4] (6 reviews^18,23,32,50,53,55^), *valued self-identity* [13.4] (14 reviews^21,24,25,27,31,37,42,43,57,58,60,62–64^), *prompts/cues* [7.1] (11 reviews^18,26,29,30,33,34,37,48,49,58,60^), *information about antecedents* [4.2] (5 reviews^32,37,41,50,58^), *behavior substitution* [8.2] (2 reviews^32,53^), and *reward (outcome)* [10.10] (1 review^53^). Reviews finding that no BCTs were found effective are listed as “none,” while reviews that did not isolate the effect of the BCT, those that did not declare effectiveness of the BCT, or those finding mixed effects are listed as “N/A” in Table 1.

**Table 1.** Summary of review articles extracted and BCTs assessed.

Citation	Substance(s) targeted	BCTs included in studies reviewed	BCTs declared effective	Number of studies included	Overall conclusions
Zhao et al., (2016)^17^	Alcohol	Self-monitoring of behavior [2.3], feedback on behavior [2.2], goal setting (behavior) [1.1], social support [3.1-3]	self-monitoring of behavior [2.3]	23	Evidence suggests that app-based interventions show promise, but more RCTs are needed to confirm effectiveness
Pugatch et al., (2018)^56^	Smoking	self-monitoring of behavior [2.3], feedback on behavior [2.2]	N/A	3	Tunneling may improve engagement and knowledge, but more research is required to support that claim
Webb et al., (2010)^18^	Alcohol, Smoking	problem solving [1.2], self-monitoring of behavior [2.3], goal setting (behavior) [1.1], action planning [1.4], feedback on behavior [2.2], prompts/cues [7.1]	goal setting (behavior) [1.1], action planning [1.4], prompts/cues [7.1], self-monitoring of behavior [2.3]	85	Findings support investing in more intensive, theory-based interventions incorporating multiple BCTs
Orr et al., (2015)^63^	Smoking	valued self-identity [13.4], reward (outcome) [10.10]	N/A	38	SMS interventions may support behavior change, though results are mixed and require further validation
Berrouiguet et al., (2016)^19^	Alcohol	self-monitoring of behavior [2.3]	self-monitoring of behavior [2.3]	36	Text messaging may enhance engagement and mental health outcomes
Donker et al., (2013)^20^	Alcohol, Smoking, Illicit Drugs	self-monitoring of behavior [2.3], feedback on behavior [2.2]	self-monitoring of behavior [2.3]	8	Evidence-based mental health apps show promise in reducing symptoms, though further validation is needed
Brown et al., (2013)^21^	Tobacco	valued self-identity [13.4], self-monitoring of behavior [2.3], feedback on behavior [2.2]	feedback on behavior [2.2]	8	There is a need for affordable, personalized, and age-appropriate tobacco cessation interventions.
Ybarra et al., (2016)^22^	Smoking	self-monitoring of behavior [2.3]	none	5	Text messaging interventions increase smoking cessation rates, though effect variability requires further study.
McCrabb et al., (2018)^23^	Alcohol, Smoking	goal setting (behavior) [1.1], action planning [1.4], feedback on behavior [2.2], self-monitoring of behavior [2.3], social support [3.1-3], problem solving [1.2]	feedback on behavior [2.2], action planning [1.4]	45	Internet-based smoking cessation interventions are effective
Mehta et al., (2010)^62^	Smoking	valued self-identity [13.4], social support [3.1-3]	valued self-identity [13.4]	10	Internet interventions reduce smoking, with valued self-identity identified as a key predictor.
Spohr et al., (2015)^24^	Smoking	self-monitoring of behavior [2.3], valued self-identity [13.4]	self-monitoring of behavior [2.3]	13	Text messaging interventions significantly improve smoking cessation outcomes.
Fjeldsoe et al., (2009)^25^	Alcohol, Smoking	self-monitoring of behavior [2.3], valued self-identity [13.4]	N/A	14	SMS interventions show positive short-term effects, though long-term efficacy remains unclear.
Palmer et al., (2018)^26^	Alcohol, Smoking	self-monitoring of behavior [2.3], feedback on behavior [2.2], problem solving [1.4], social support [3.1-3], prompts/cues [7.1], goal setting (behavior) [1.1]	N/A	72	SMS-based smoking cessation support increases quit rates.
Oosterveen et al., (2017)^27^	Alcohol, Smoking	valued self-identity [13.4], self- monitoring of outcomes of behavior [2.4], feedback on behavior [2.2]	N/A	45	eHealth SNAPO interventions show short-term efficacy, with limited evidence on long-term outcomes.
Hutton et al., (2020)^28^	Alcohol	feedback on behavior [2.2], self-monitoring of behavior [2.3], goal setting (behavior) [1.1]	N/A	18	mHealth interventions provide an accessible and cost-effective method for youth health promotion.
Tofighi et al., (2017)^29^	Alcohol, Illicit Drugs	self-monitoring of behavior [2.3], prompts/cues [7.1], social support 3.1-3], goal setting (behavior) [1.1], feedback on behavior [2.2]	N/A	11	Text messaging interventions are feasible and acceptable for substance use treatment.
Quanbeck et al., (2014)^30^	Alcohol	goal setting (behavior) 1.1], feedback on behavior [2.2], prompts/cues [7.1], self-monitoring of behavior [2.3]	N/A	14	mHealth systems vary widely in complexity, from simple reminders to comprehensive recovery tools.
Dick et al., (2019)^57^	Illicit Drugs	feedback on behavior [2.2], valued self-identity [13.4]	feedback on behavior [2.2]	8	Digital interventions may modestly reduce harm from substance misuse.
Kong et al., (2014)^58^	Smoking	information about antecedents [4.2], feedback on behavior [2.2], social support [3.1-3], goal setting (behavior) [1.1], prompts/cues [7.1], valued self-identity [13.4]	N/A	22	Connected interventions show positive effects on SUD recovery and high acceptability.
Scott-Sheldon et al., (2016)^31^	Smoking	feedback on behavior [2.2], goal setting (behavior) [1.1], valued self-identity [13.4], self-monitoring of behavior [2.3], social support	N/A	20	Text messaging interventions are highly effective for smoking cessation and scalable at low cost.
Nesvag et al., (2018)^65^	Alcohol, Cannabis	feedback on behavior [2.2], social-support [3.1-3], self-monitoring of behavior [2.3]	N/A	43	Digital interventions are feasible but show inconsistent effectiveness in SUD recovery outcomes.
Kaner et al., (2017)^32^	Alcohol	feedback on behavior [2.2], goal setting (behavior) [1.1], self-monitoring of behavior [2.3], action planning [1.4], information about antecedents [4.2], social support [3.1-3], problem solving [1.2], behavior substitution [8.2]	behavior substitution [8.2], problem solving [1.2],	57	Digital interventions moderately reduce alcohol consumption in the short term.
Getty et al., (2019)^59^	Alcohol, Tobacco	feedback on behavior [2.2]	N/A	6	Mobile-delivered contingency management shows potential for enhancing SUD treatment.
Staiger et al., (2020)^33^	Alcohol, Tobacco, Illicit Drugs	feedback on behavior [2.2], self-monitoring of behavior [2.3], action planning [1.4], information about antecedents [4.2],	none	20	App-based interventions should be compared to equivalent non-app formats to determine relative efficacy.
O’rourke et al., (2016)^34^	Alcohol	feedback on behavior [2.2], prompts/cues [7.1], self-monitoring of behavior [2.3]	feedback on behavior [2.2], prompts/cues [7.1]	11	Technology-based interventions offer flexibility that is particularly appealing to young adults.
Ashford et al., (2019)^35^	Alcohol, Illicit Drugs	goal setting (behavior) [1.1], self-monitoring of behavior [2.3], social support [3.1-3]	self-monitoring of behavior [2.3]	22	Digital recovery services show potential but currently lack strong evidence of effectiveness.
Gonzalez et al., (2021)^36^	Alcohol, Smoking	self-monitoring of behavior [2.3], feedback on behavior [2.2]	N/A	23	Cultural tailoring in mHealth is increasing, though mechanisms remain poorly understood.
Vodopivec-Jamsek et al., (2012)^37^	Alcohol, Tobacco	self-monitoring of behavior [2.3], information about antecedents [4.2], valued self-identity [13.4], feedback on behavior [2.2], prompts/cues [7.1]	none	4	Mobile messaging improves preventive health behaviors, though effects vary.
Giroux et al., (2017)^38^	Alcohol, Illicit Drugs	self-monitoring of behavior [2.3], feedback on behavior [2.2]	N/A	18	Online psychological interventions largely reflect traditional self-administered approaches.
Holmes et al., (2018)^39^	Alcohol, Smoking	self-monitoring of behavior [2.3]	N/A	6	Digital tools may improve access to mental health care, though evidence remains limited.
Hoeppner et al., (2017)^40^	Alcohol	self-monitoring of behavior [2.3], goal setting (behavior) [1.1], feedback on behavior [2.2]	self-monitoring of behavior [2.3], feedback on behavior [2.2]	266	Smartphone apps for alcohol use show potential public health benefits.
Carreiro et al., (2020)^41^	Alcohol	self-monitoring of behavior [2.3], feedback on behavior [2.2], information about antecedents [4.2]	feedback on behavior [2.2]	32	Digital interventions are associated with improved SUD recovery outcomes.
Sawares et al., (2017)^42^	Alcohol	valued self-identity [13.4], self-monitoring of behavior [2.3]	N/A	10	User engagement is critical for achieving behavior change in digital interventions.
Head et al., (2013)^64^	Smoking	valued self-identity [13.4]	N/A	19	Personalized interventions are significantly associated with intervention effectiveness
Hall et al., (2015)^60^	Smoking	feedback on behavior [2.2], prompts/cues [7.1], valued self-identity [13.4]	none	15	Evidence on optimal intervention characteristics is limited; further research is needed.
Humphreys et al., (2021)^44^	Alcohol	problem solving [1.2], feedback on behavior [2.2], self-monitoring of outcomes of behavior [2.2]	problem solving [1.2], feedback on behavior [2.2], self-monitoring of outcomes of behavior [2.2]	45	Identifying common BCTs and controlling studies for quality and effectiveness can inform development of transdiagnostic interventions.
Champion et al., (2019)^45^	Alcohol, Smoking	self-monitoring of behavior [2.3], goal setting (behavior) [1.1], self-monitoring of behavior [2.3], feedback on behavior [2.2]	N/A	22	eHealth school-based interventions show small but positive effects on health behaviors.
Hou et al., (2014)^46^	Alcohol, Tobacco	feedback on behavior [2.2], goal setting (behavior) [1.1], and self-monitoring of behavior [2.3]	feedback on behavior [2.2], self-monitoring of behavior [2.3]	38	Web-based interventions are generally effective in improving targeted health outcomes.
Tong et al., (2021)^47^	Alcohol, Smoking	feedback on behavior [2.2], self-monitoring of behavior [2.3], goal setting (behavior) [1.1]	self-monitoring of behavior [2.3]	39	Personalized mobile interventions show early promise but remain underdeveloped.
Kohl et al., (2013)^61^	Alcohol, Smoking	feedback on behavior [2.2], goal setting (behavior) [1.1]	N/A	41	Digital interventions often produce small and unsustained behavior change effects.
Gold et al., (2021)^48^	Alcohol, Smoking	feedback on behavior [2.2], self-monitoring of behavior [2.3], prompts/cues [7.1]	N/A	92	Digital interventions yield small positive effects, potentially limited by adherence issues.
Piette et al., (2017)^49^	Smoking	feedback on behavior [2.2], prompts/cues [7.1], self-monitoring of behavior [2.3], goal setting (behavior) [1.1]	N/A	N/A	Interactive voice response interventions improve cardiovascular risk factors and disease management.
Furness et al., (2020)^50^	Alcohol, Smoking	goal setting (behavior) [1.1], self-monitoring of behavior [2.3], information about antecedents [4.2], problem solving [1.2], action planning [1.4], feedback on behavior [2.2]	N/A	24	eHealth interventions benefit cancer patients, though delivery mode effects are unclear.
Milne-Ives et al., (2020)^51^	Alcohol, Illicit Drugs	goal setting (behavior) [1.1], feedback on behavior [2.2], self-monitoring of behavior [2.3]	N/A	52	Greater evaluation of individual and combined BCTs is needed to improve app effectiveness.
Akinosun et al., (2021)^52^	Alcohol, Smoking	social support [3.1-3], goal setting (behavior) [1.1]	social support [3.1-3]	25	Digital interventions show limited impact on some cardiovascular risk factors.
Armanasco et al., (2017)^43^	Alcohol, Smoking	self-monitoring of behavior [2.3], valued self-identity [13.4], reward(outcome) [10.10], goal setting (behavior) [1.1]	N/A	35	Further behavior-specific and quantitative research is recommended.
Edwards et al., (2016)^53^	Alcohol, Smoking	goal setting (behavior) [1.1], self-monitoring of behavior [2.3], feedback on behavior [2.2], reward (outcome) [10.10], social support [3.1-3], action planning [1.4]	N/A	55	Stronger collaboration across disciplines is needed to maximize intervention impact.
Morrison et al., (2012)^54^	Smoking	social support [3.1-3]	N/A	52	More research is needed on user-initiated and self-management features.
Young et al., (2018)^55^	Alcohol, Smoking	self-monitoring of behavior [2.3], social support [3.1-3], goal setting (behavior) [1.1], action planning [1.4], problem solving [1.2]	goal setting (behavior) [1.1]	7	Online lifestyle interventions may improve depressive symptoms when targeting behavior change.

### Review findings

Six reviews^17,18,20,24,40,44^ were able to isolate the effects of *self-monitoring of behavior* [2.3], and all six concluded that this BCT was found effective for positive clinical outcomes, including smoking reduction and reduction of alcohol consumption. Three additional reviews showed mixed results after being unable to fully isolate the effects of [2.3] among their included studies, and thus could not render strong conclusions.^19,35,56^ Additionally, three reviews^22,33,37^ found [2.3] ineffective. Only four reviews^34,40,44,47^ were able to isolate the positive effects of *feedback on behavior* [2.2], finding the BCT effective for reducing consumption of alcohol and tobacco use, and three additional reviews^23,41,57^ showed mixed results. Two reviews^33,37^ found that [2.2] was ineffective. After self-monitoring and feedback, the next most studied BCT in these reviews was goal setting for behavior [1.1],^17,18,23,26,28–32,35,40,43,45–47,49–53,55,58,61^ though only one review^55^ was able to isolate the effects of the BCT, concluding that this BCT was found effective in reducing alcohol use and smoking. One additional review^18^ found mixed results. *Social support* [3.1-3] was also commonly studied and evaluated in 13 reviews,^17,23,26,29,31,32,35,52–55,58,62^ though none reported clear evidence that these BCTs were effective to promote substance use reduction.

Another common BCT was *prompts/cues* [7.1], studied in 11^18,26,29,30,33,34,37,48,49,58,60^ reviews, with one review^34^ finding *prompts/cues* [7.1] effective for a reduction in alcohol use and another review^18^ finding mixed results. One review found the BCT ineffective.^37^ *Valued self-identity* [13.4], was evaluated in 14 reviews,^21,24,25,27,31,37,42,43,57,58,60,62–64^ with only one review^62^ finding the BCT effective for promoting smoking reduction and one review finding the BCT ineffective for alcohol and tobacco cessation.^37^ While *information about antecedents* [4.2] was studied in five reviews,^32,37,41,50,58^ it was not found individually effective in any reviews, but it was found ineffective in two reviews.^33,37^ Another BCT included in the reviews was *problem solving* [1.2], used in seven reviews,^18,23,26,32,44,50,55^ with two^32,44^ of the reviews that included it found that it was effective, though without differentiating effectiveness between behaviors. *Action planning* [1.4], used in six reviews,^18,23,32,50,53,55^ was found to have mixed results in two reviews,^18,23^ however, it was not reported as individually effective in any review. It was reported as ineffective in one review.^33^ *Behavior substitution* [8.2] was used in two reviews,^32,53^ and was found effective for reducing alcohol consumption in 50% of the reviews in which it was included. *Reward on outcome* [10.10] was studied in three reviews^43,53,63^ however, none were able to isolate the effect of the BCT. The total number of reviews including and evaluating each BCT is shown in Table 2.

**Table 2.** Total number of reviews in which each BCT was evaluated, found effective, or results were mixed.

BCT	Number of reviews reported	Number of reviews where unique effect of BCT was evaluated	Number of reviews where BCT was found effective	Number of reviews where BCT had mixed results	Number of reviews where BCT was found ineffective
Self-Monitoring of behavior	40	12	6	3	3
Feedback on behavior	34	9	4	3	2
Goal Setting (behavior)	23	2	1	1	0
Social Support	13	0	0	0	0
Prompts/cues	11	3	1	1	1
Valued Self-Identity	14	2	1	0	1
Information About Antecedents	5	2	0	0	2
Problem Solving	7	2	2	0	0
Action Planning	6	3	0	2	1
Reward (outcome)	1	0	0	0	0
Behavior Substitution	2	1	1	0	0

## Summary of results

While *feedback on behavior* [2.2], *self-monitoring of behavior* [2.3], and *goal setting (behavior)* [1.1] were evaluated in the most reviews, *prompts/cues* [7.1], and *behavior substitution* [8.2] were effective in the largest proportions of the reviews that included them. *Problem solving* [1.2] and *action planning* [1.4] had the next largest proportions, while *self-monitoring of behavior* [2.3], *feedback on behavior* [2.2], *goal setting (behavior)* [1.1], and *valued self-identity* [13.4] had the lowest non-zero proportions for effectiveness.

## Discussion

Overall, most of the systematic reviews and meta-analyses included in this synthesis yielded mixed or inconclusive results and declared that further research is needed to better characterize BCT effectiveness for preventing substance use in JITAIs. Most of the reviews in this paper yielded mixed results for the effects of individual BCTs on positive behavior change, due to variations between results in individual studies, where only some studies within each review were able to isolate the effects of the BCTs. This limited the ability to determine conclusive results for the effects of BCTs; however, it helps clarify where evidence is beginning to emerge and highlights what steps need to be taken in the future to reach more conclusive results.

Despite these mixed findings, this synthesis provides a clearer picture of how BCTs are currently being implemented and evaluated in digital substance use interventions. While several techniques, such as *feedback on behavior* [2.2], *self-monitoring of behavior* [2.3], and *goal setting (behavior)* [1.1] were evaluated in the most reviews, *prompts/cues* [7.1] and *behavior substitution* [8.2] appeared most promising. These finding suggest that some BCTs may deserve more attention in future intervention design and evaluation. Ybarra et al. suggests that the next steps for smoking cessation, in particular, is to determine if the just-in-time components, like the text-messages themselves, are sufficient on their own or if other BCT components, such as setting a quit day and preparing, need to be paired with them.^22^ In addition, they suggest that future studies should compare text messaging interventions to other controls that do not require an in-person component, such as telephone quit lines, which would provide context for the relative impact of mHealth interventions.^22^ Spohr et al. provides a different approach, suggesting that prioritizing the development of adaptively tailored programming for text message interventions in smoking cessation, especially with the incorporation of ecological momentary assessment (EMA) would be beneficial to mHealth research.^24^ EMA, especially when used in conjunction with behavioral interventions, allows real-time assessment of cue-induced craving, facilitating proactive measures to prevent relapse. The reviews also acknowledge potential biases in their studies, but generally, most reviews declare a low risk of bias. Additionally, many studies within the reviews utilized randomized controlled trials, which allow for a more systematic approach to analysis that can be more easily replicated.

A major limitation of our synthesis was the inability to conduct substance-specific analysis. Of the 49 studies included in this synthesis, 36 focused on alcohol use as the primary health behavior, while several others grouped multiple substances, such as alcohol use and smoking use, together. Because of this, isolated analysis on specific substances was infeasible. The individual reviews included in our synthesis also had some limitations, such as including a small number of studies or a small number of participants in each individual study. Many reviews focused on the effects of the delivery method (e.g., the app itself) rather than the effect of the BCTs used, which is not applicable to our review. Incomplete reporting of BCTs was a considerable confounding factor across many such reviews and is not easily addressable without systematic reconsideration in reporting of systematic reviews and meta-analyses and highlights the need for interventionists to attempt to isolate the effects of key BCTs whenever possible. Other reviews evaluated which BCTs were used most often without considering the effectiveness of those BCTs. Another common limitation among the systematic reviews was that the studies analyzed were too heterogenous with respect to target outcomes for a meta-analysis to be conducted. Many studies focused on the effectiveness of the intervention, rather than the effectiveness of the BCT, while still acknowledging the use of BCTs. For example, Scott-Sheldon et al.^31^ and Palmer et al.^26^ found that text messaging interventions were correlated with increased smoking cessation rates, indicating that such JITAIs show promise for improving substance use outcomes. While these findings demonstrate the potential effectiveness of digital interventions, they do not isolate the effects of individual BCTs contributing to these outcomes. Additionally, since these conclusions were drawn across reviews with various study designs, samples, and outcome measures, some variability in effectiveness findings is expected and reflects the nature of such a synthesis. A few reviews acknowledge the fact that there was a wide variability in definitions and in inclusion criteria, so it was difficult to accurately compare across studies. Moving forward, it will be helpful for future interventions to adopt similar reporting measures and outcomes to promote comparability and enable more definitive conclusions to be conferred.

One major limitation of our study was that it was difficult to compare the usage of BCTs across multiple systematic reviews and meta-analyses because there was a lack of uniformity. Some reviews did not state explicitly which BCTs were used, and instead, they were described in the methods. In these cases, trained coders certified from the BCT taxonomy retroactively coded which BCT was being described, provided sufficient description to meet criteria. While multiple steps were taken to ensure as much accuracy as possible in these codings, there is likely error present. In the future, it would be beneficial for studies to specifically identify which BCTs are used in their interventions, perhaps as a supplementary table or file, to better facilitate systematic comparison and move the field forward. We recommend that researchers consider factorial designs or other innovative trial designs to pinpoint the active ingredients of interventions.

## Conclusion

While few definitive conclusions could be made from the review of 49 systematic reviews and meta-analyses focusing on substance use, there are promising results that clearly set up the path for future research of the use of BCTs in JITAIs for substance abuse cessation and relapse prevention. Although there is evidence suggesting that social support, feedback on behavior, and self-monitoring of outcomes of behavior are effective for relapse prevention, these results are mixed within reviews, so further research should evaluate the *context* in which these BCTs are most effective. Notably, this synthesis sheds light on less commonly studied BCTs such as *prompts/cues* [7.1] and *behavior substitution* [8.2], which are promising avenues for further exploration. Beyond echoing the call for more investigation, this synthesis serves as a roadmap, pinpointing specific limitations in each step in the formulation of a systematic review/meta-analysis and providing suggestions on how to improve upon them for future research. We recognize the complexities in drawing conclusions from the existing literature and advocate for a more refined approach in future research. Rather than offering a single definitive answer, this synthesis clarifies which BCTs are most frequently used across digital substance use interventions and which appear most consistently associated with positive outcomes among prior reviews. By doing so, we provide a reference point for future intervention development and highlight areas where more rigorous research is needed to better understand the active ingredients of digital substance use interventions.

## Appendix

### Appendix A: search terms

(((Program[tiab] OR Intervention[tiab] OR interventional[tiab] OR “internet-based intervention”[MeSH] OR “Randomized Controlled Trials as Topic”[MeSH])
AND
(“cell phone”[MeSH] OR “mobile applications”[MeSH] OR “mobile applications”[tiab] OR smartphone[tiab] OR “smart phone”[tiab] OR mobile[tiab] OR “mHealth”[tiab] OR “m-health”[tiab] OR “e-health”[tiab] OR “eHealth”[tiab] OR tablet[tiab] OR “text messag*”[tiab] OR PDA[tiab] OR “personal digital assistant”[tiab]))
AND
((diet[tiab] OR “Diet”[MeSH] OR “dietary intake”[tiab] OR “dietary intakes”[tiab] OR calorie*[tiab] OR “caloric intake”[tiab] OR “food intake”[tiab] OR “energy intake”[MeSH] OR nutrition[tiab] OR “caloric restriction”[MeSH] OR eating[MeSH] OR “binge eating”[tiab] OR “binge-eating disorder”[MeSH] OR “food addiction”[MeSH] OR “substance-related disorders”[MeSH] OR “controlled substances”[MeSH] OR “substance use”[tiab] OR “substance misuse”[tiab] OR smok*[tiab] OR vape[tiab] OR vaping[tiab] OR marijuana[tiab] OR “marijuana use”[MeSH] OR “alcohol drinking”[MeSH] OR “alcohol-related disorders”[MeSH] OR “drinking behavior”[MeSH] OR “binge drinking”[MeSH] OR “drinking”[MeSH] OR alcohol[tiab] OR drink*[tiab] OR cocaine[tiab] OR heroin[tiab] OR amphetamine[MeSH] OR amphetamine[tiab] OR “drug abuse”[tiab] OR “drug use”[tiab] OR “substance abuse, oral”[MeSH] OR “substance abuse, intravenous”[MeSH] OR “substance abuse”[tiab] OR opioid[tiab] OR tobacco[tiab] OR “tobacco use”[MeSH] OR “tobacco smoking”[MeSH] OR “tobacco use cessation”[MeSH] OR nicotine[tiab] OR cigarette*[tiab] OR ecstasy[tiab] OR MDMA[tiab])))
AND
((BCT[tiab] OR “behavior change”[tiab] OR “behaviour change”[tiab] OR “behavior change technique”[tiab] OR “behaviour change technique”[tiab] OR “Goal setting”[tiab] OR goal*[tiab] OR “Problem solving”[tiab] OR “Action planning”[tiab] OR “behavior goal*”[tiab] OR “behavioral goal*”[tiab] OR “behaviour goal*”[tiab] OR “behavioural goal*”[tiab] OR “behavioral contract*”[tiab] OR “behavioural contract*”[tiab] OR Feedback[tiab] OR “Self-monitoring”[tiab] OR “Social support”[tiab] OR “Social comparison”[tiab] OR “Prompts/cues”[tiab] OR “prompts”[tiab] OR “cues”[tiab] OR “prompts and cues”[tiab] OR “Behavior substitution”[tiab] OR “Behaviour substitution”[tiab] OR “Self-reward”[tiab] OR Restructuring[tiab] OR restructure[tiab] OR “past success”[tiab])).
